# Alternative Antibiotics in Dentistry: Antimicrobial Peptides

**DOI:** 10.3390/pharmaceutics14081679

**Published:** 2022-08-12

**Authors:** Alexandra Griffith, Akilah Mateen, Kenneth Markowitz, Steven R. Singer, Carla Cugini, Emi Shimizu, Gregory R. Wiedman, Vivek Kumar

**Affiliations:** 1 Department of Biomedical Engineering, New Jersey Institute of Technology, Newark, NJ 07102, USA; 2 Department of Chemistry and Biochemistry, Seton Hall University, South Orange, NJ 07079, USA; 3 Department of Oral Biology, Rutgers School of Dental Medicine, Newark, NJ 07103, USA; 4 Department of Diagnostic Sciences, Rutgers School of Dental Medicine, Newark, NJ 07103, USA; 5 Department of Endodontics, Rutgers School of Dental Medicine, Newark, NJ 07103, USA; 6 Department of Biology, New Jersey Institute of Technology, Newark, NJ 07102, USA; 7 Department of Chemical Engineering, New Jersey Institute of Technology, Newark, NJ 07102, USA

**Keywords:** antibiotic resistance, anti-microbial peptides, AMPs, microbiome, oral infections

## Abstract

The rise of antibiotic resistant bacteria due to overuse and misuse of antibiotics in medicine and dentistry is a growing concern. New approaches are needed to combat antibiotic resistant (AR) bacterial infections. There are a number of methods available and in development to address AR infections. Dentists conventionally use chemicals such as chlorohexidine and calcium hydroxide to kill oral bacteria, with many groups recently developing more biocompatible antimicrobial peptides (AMPs) for use in the oral cavity. AMPs are promising candidates in the treatment of (oral) infections. Also known as host defense peptides, AMPs have been isolated from animals across all kingdoms of life and play an integral role in the innate immunity of both prokaryotic and eukaryotic organisms by responding to pathogens. Despite progress over the last four decades, there are only a few AMPs approved for clinical use. This review summarizes an Introduction to Oral Microbiome and Oral Infections, Traditional Antibiotics and Alternatives & Antimicrobial Peptides. There is a focus on cationic AMP characteristics and mechanisms of actions, and an overview of animal-derived natural and synthetic AMPs, as well as observed microbial resistance.

## 1. Introduction to Oral Microbiome and Oral Infections

The oral cavity is the entrance to the digestive track, with each region, including the mouth, having its own unique niche and thus microbial signature [1,2]. Well over 700 distinct microbial species have been identified in the human oral microbiome, with every person having their own combination of these microorganisms [3,4]. An individual’s unique oral microbiome was historically thought to form from a sterile environment at birth, though recent clinical data has shown that there is exposure to microbes in utero without overt infection [5,6,7,8,9,10]. The oral cavity is then inoculated by the microorganisms that will make up that individual’s oral microbiome, starting with their first feeding and gradually continuing with specific events, such as tooth eruption, that allow particular species to colonize specific tissue niches [11,12]. As the mouth develops and the types of tissues available for bacterial attachment increase, the diversity of bacterial species in the oral cavity increases [3]. The microorganisms biogeography in their respective niches in the oral cavity are very specific to the tissue type, location, and presence of other microbes [13].

Of the many tissue types in the oral cavity, teeth are one of the only exposed non-shedding surfaces in the body that allows regular colonization by microorganisms, forming biofilms that protect the colony, allowing bacteria to flourish and proliferate [14,15]. This allows the mouth to act as a reservoir for harmful bacteria that, when shed from their biofilm colony, can travel to other areas of the body to cause second site infections [16,17,18], making it critical to treat infections in the oral cavity.

**Figure 1:**
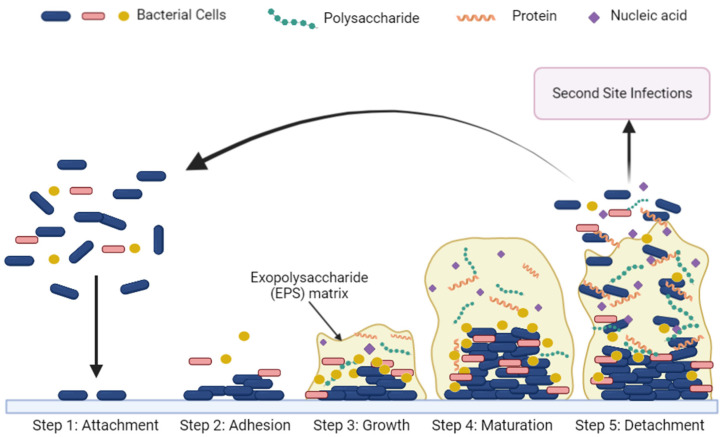
Progression of biofilm formation in the oral cavity. In the attachment stage (Step 1), planktonic cells begin to adhere to a surface. A monolayer of planktonic cells accumulates on the surface during the adhesion stage (Step 2). During the growth stage (Step 3), sessile cells begin to excrete an exopolysaccharide matrix containing proteins, polysaccharides (carbohydrates) and nucleic acids that surround the cells. The EPS matrix assumes a mushroom cloud shape in the maturation state (Step 4). Finally, planktonic cells are released from the EPS matrix during the detachment stage (Step 5), allowing for dispersal and colonization of new sites. (Created with BioRender, accessed on 1 August 2022).

The oral bacteria generally form a multispecies biofilm on the hard and soft tissue referred to as plaque. Biofilms consist of bacterial cells enmeshed in an extracellular matrix containing extracellular polysaccharides (EPS), proteins, and extracellular-bacterial DNA [19,20] (Figure 1). Competitive as well as cooperative interspecies interactions shape the microbial community in plaque where some species secrete factors that are toxic to others and where metabolic byproducts of one species may be vital nutrients for other organisms [21,22,23]. Along with available oxygen, the cooperative metabolism results in not a homogenous biogeography but a complex species–specific cell–cell architecture and organization [24,25].

Bacteria that form or participate in a biofilm are protected from the toxic effects of antimicrobials and antibiotics [26,27]. This is particularly true of organisms situated deep within the biofilm. Minimum inhibitory concentration (MIC), disk diffusion assay or other tests of antibiotic performance do not always reflect the protective properties that growth in biofilms afford the bacteria, as many of these tests are performed on planktonically grown cells [28,29,30]. Other methods, such as minimum biofilm eliminating concentration (MBEC) and 2,3-bis-(2-methoxy-4-nitro-5-sulfophenyl)-2H-tetrazolium-5-carboxanilide (XTT) assays, should be used instead [31,32,33]. The issue of measuring antibiotic performance is particularly true of organisms situated deep within the biofilm, for which a minimum biofilm inhibitory concentration (MBIC) assay will be most applicable, given a diffusion gradient exists within the biofilms. Numerous studies have shown that when exposed to antibiotics and antimicrobials, organisms grown in biofilms have a lower antimicrobial sensitivity and many organisms will experience concentrations below the planktonic MIC [34,35]. This can encourage the selection of resistant organisms in the population through a number of different mechanisms. This selection has been shown to enhance the expression of biofilm-formation genes in the surviving organisms [36]. Another mechanism is the ability of bacteria to exchange genes within species and between species, which allows resistant biofilms to be an important source of antibiotic resistant organisms that can endanger human and animal populations [37].

Dental biofilms are influenced by our physiology, behavior and diet [14,38]. Although the exact contribution of many oral hygiene practices to the occurrence of dental diseases is unclear, what is undisputed is that the accumulation of plaque can cause a shift in the microbial population favoring Gram-negative anaerobic pathobiont species that are present at low levels in health [39,40]. This shift in the plaque composition that favors disease-associated bacteria is called dysbiosis [41]. In contrast, maintaining a thin plaque will promote colonization of those bacteria that first colonize exposed tooth surfaces, which are generally considered commensals except that they can sometimes be associated with particular diseases, such as infective endocarditis.

Dysbiosis is a hallmark of periodontal disease. *Porphyromonas gingivalis* (Pg), *Fusobacterium nucleatum* (Fn) and other bacteria associated with periodontal disease can be recovered at low levels from patients with periodontal health [42,43]. Many Gram-negative anaerobic species, such as Pg, present virulence factors that cause the bacteria to be inflammophilic, driving the progression of gingivitis to periodontitis [44]. The inflammatory process that follows when dysbiosis has occurred can eventually trigger bone loss. This bone loss weakening the support of the teeth is the defining feature of periodontal disease [45,46]. In addition, these periodontal related oral infections can lead to secondary systemic effects, such as cardiovascular disease, respiratory infections, or infective endocarditis [17,47].

**Figure 2:**
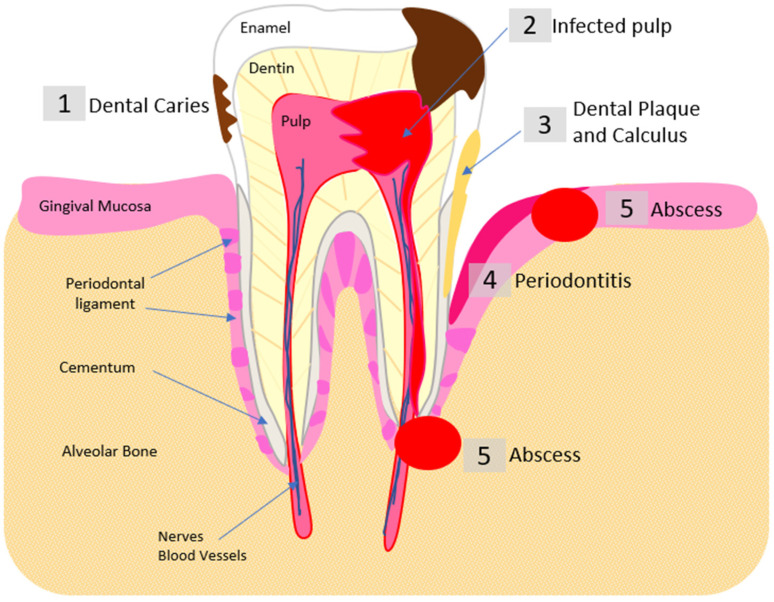
Localization of bacterial oral pathogenesis. (1) Dental Caries: tooth enamel dissolved by acid produced by specific bacteria (e.g., *Mutans streptococci*, *Lactobacilli*). (2) Dental Pulp Infection: necrotic pulp tissues infected by microorganisms causing pulp inflammation and eventual necrosis. (3) Plaque and Calculus: biofilms form on non-shedding surfaces of the teeth; formation leads to caries and periodontal inflammation. (4) Periodontitis: dysbiotic bacterial infections cause inflammation that destroys periodontal ligament and bone. (5) Abscess: periodontal and periapical abscesses are pockets of pus caused by bacterial infection.

Dental caries is also a disease marked by localized biofilm dysbiosis. The physical manifestation of this disease is caused by acid dissolution of the outer tooth mineral, enamel (Figure 2) [48]. Early colonizers associated with dental caries such as *Streptococcus mutans* and *Neisseria bacilliformis* are acid producing and acid tolerant [48,49,50]. Sugars are the substrate these bacteria use for acid production by fermentation, creating an acidic environment more suited to these acidogenic and aciduric bacteria. Diets rich in simple sugars favor these species since low pH levels in the plaque inhibit the growth of many other bacteria that are considered members of the healthy flora [51]. Saliva buffers the bacterially-generated acids restoring the plaque pH to normal levels following sugar ingestion [52]. Persons who have low saliva flow are at great risk of caries since these acids are not buffered [53]. This also plays a role in the plaque composition since the maintained low pH environment favors *Streptococcus mutans*, *Actinomyces naeslundii* and other acid tolerant organisms [54]. Since many medications that older individuals use adversely affect saliva flow, caries particularly on susceptible root surfaces are common in the elderly [55,56].

**Figure 3:**
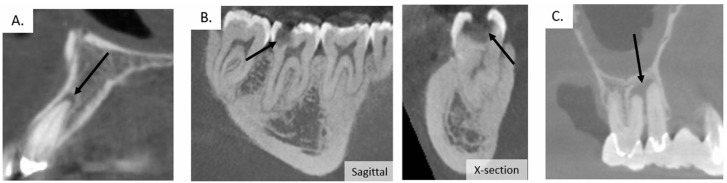
Cone beam computed tomography (CBCT) images detect the presence of apical inflammatory lesions. This type of empirical evidence for oral infections is used as a method of deciding treatment course by dentists. (**A**) CBCT sagittal image shows an apical inflammatory lesion as a darker grey pocket surrounding the apex of tooth. (**B**) CBCT images show dental caries from sagittal and cross-sectional views. (**C**) Sagittal CBCT image shows large apical inflammatory lesion between the apices of adjacent teeth. From the author’s collection.

As carious lesions develop, cavitated lesions develop that invade the dentin, the deeper less mineralized tissue of the tooth, and eventually the dental pulp, the small area of soft tissue inside the tooth (Figure 2) [39]. The dental pulp contains nerves, blood vessels and cells active in the host defense. The pulp space is referred to as the root canal. Deep caries has a diverse bacterial population which can contain *Lactobacillus*, *Prevotella*, and *Treponema* species as well as several types of anaerobes, such as *Porphyromonas gingivalis* (Pg), which is also associated with periodontal disease [42,57,58]. Infections of the root canal results from deep caries, trauma to the tooth and poorly performed dental procedures that injure the pulp. If a root canal infection is not treated, it can lead to periapical abscesses causing systemic health issues that require hospitalization [59,60]. These endodontic infections of the root canal space and dental pulp tissue can lead to apical periodontitis, periapical abscesses and eventually tooth loss, which can be easily visualized using cone-beam computed tomography or standard intraoral and panoramic radiographs (Figure 3) [58]. Root canal treatment (endodontic therapy) cleans and sterilizes the root canal space and fills it with an inert material. Occasionally infection persist despite root canal treatment. *Enterococcus faecalis* has been observed to be a species commonly cultured in persistent root canal infections [58,61,62]. Because of this, Oral health is intricately connected to systemic health [63,64,65].

In addition to the bacterial presence, various fungi are commonly present in the oral cavity. *Candida albicans* is the principal cause of fungal infection in the oral cavity. In general, clinical manifestations of Candida infections in the human host range from irritating red and white lesions on mucosal surfaces to opportunistic invasive or even life-threatening systemic infections in the immunosuppressed. *Candida* can grow on mucosal surfaces as yeast or produce hyphae and invade tissue. Hyphae formation and the production of various proteolytic enzymes are important virulence factors [66]. Dysbiosis also plays an important role in Candida infection [67]. Reducing the resident commensal protective bacterial population with antibiotic use predisposes the mucosal surfaces to Candida overgrowth, such as what is seen thrush [68]. A commonly problem seen in oral cavity is driven by lack of adequate denture hygiene measures, which provides an ideal environment for Candida growth in the space between dentures and the oral mucosa [69]. In addition, patients whose bite has collapsed due to tooth loss, Candida can grow in the moist environment in the corners of the mouth. Since *Candida albicans*, is an acid tolerant organism [70] like *Streptococcus mutans*, current research has examined the possible relationship between these phylogenetically diverse organisms in the ecology of severe caries [71,72].

Antibiotics are used by clinicians to treat bacterial infections in the oral cavity: dental abscesses, sinusitis causing tooth pain, pericoronitis and acute gingivitis and periodontitis [73,74,75,76]. In general practice, clinicians do not identify the bacterium or bacteria responsible for the oral infection by culturing the pus or exudate. Because of this, most antibiotics prescribed by dentists are broad spectrum as they do not target the specific bacteria responsible for the infection [77]. There has been an increase in strains of bacteria in the oral cavity that are resistant to antibiotics used by clinicians [78]. As antibiotic resistance increases, existing antibiotics become less effective. In dentistry specifically, more careful prescription of antibiotics is needed, which critically impacts systemic health and antibiotic resistance (AR) [79,80].

What is apparent in this brief overview of oral bacterial and fungal infection is the importance of environmental and host factors and the microbial ecology in causing dysbiosis. Antibiotics target specific pathogens with the aim of eliminating them from sites of infection. Since antibiotics have many undesirable actions, including indiscriminate microbial killing favoring the growth of resistant organisms, their use is largely restricted to the treatment of severe infection. Technologies that are more selective can be used to reverse dysbiosis and restore a healthy microbial balance.

## 2. Traditional Antibiotics and Alternatives

Antibiotics have saved millions of lives since their discovery by Alexander Fleming in 1928; however, their effectiveness is in jeopardy due to the development of antibiotic resistant bacterial strains [81]. As of 2022, antibiotic resistant bacterial infections killed more people annually than malaria or HIV/AIDS [82]. The over prescription of antibiotics in unnecessary circumstances in the healthcare setting is seen as a main cause for the increase in the prevalence of antibiotic resistant bacterial strains [83]. This over-prescription has been noted especially in the dental field, where antibiotics are routinely prescribed for many treatment forms, such as chronic apical inflammation [84], pulpitis [85] and periodontal infection [86,87]. In addition, antibiotic prophylaxis in the absence of infection contributes to the issue of antibiotic resistance [88,89]. Regulatory groups and several studies recommend that, as a field, dentists prescribe antibiotics more carefully as well as looking into alternative treatment methods [79,85,90,91,92].

### 2.1. Overview of Mechanism of Action and Resistance

Antibiotics prescribed for dental infections function using one of a few key target mechanisms: inhibiting the bacterial cell’s ability to synthesize the cell wall, nucleic acids or proteins [93]. Antibiotics such as penicillin target the cell wall by removing available material used for cell wall construction, causing a disruption leading to bacterial lysis. Antibiotics such as clindamycin and tetracyclines inhibit protein synthesis using specific targeting of either the 50 S or 30 S ribosomal subunit found only in bacteria and not in eukaryotic cells. Sulfonamides and quinolones function by disrupting nucleic acid synthesis [94].

Resistance in bacteria can develop against these antibiotics that target nucleic acid, protein or cell wall synthesis. The bacterial cell wall can become less penetrable to these antibiotics by decreasing the amount of porin channels through the outer membrane that allow the antibiotic to enter the cell [94], or the bacterial cell wall may develop reduced uptake or active extrusion of the drug, causing a less effective response to the antibiotic by failure of the antibiotic to reach the target site [95]. Penicillin binding proteins (PBPs) are a common target of antibiotics that disrupt cell wall synthesis. Bacteria develop resistance to the antibiotics that target these PBPs by developing an altered protein that fails to bind to penicillin [96]. PBPs are essential to the production of the bacterial cell wall. They allow the bacteria to increase or replace its existing cell wall or divide into daughter cells by cross linking cell wall peptidoglycans. Antibiotics targeting PBPs disrupt this process [94,97]. Antibiotics that alter or halt protein translation can lose their effectiveness as bacteria develop resistance when the bacterial ribosome is altered either in the 50 S or the 30 S subunit. Decreased penetration and uptake of the antibiotic in the resistant bacteria make the drug less effective over time, and specific mutations of the effective binding site occur in the resistant strains [94].

### 2.2. Opportunities for Alternative Approaches

Fortunately, the oral tissues are accessible and various antimicrobial agents can be applied either by the dentist as part of treatment or by the patient at home. Dentists have employed local delivery systems to administer antibiotics and other agents to the periodontium [98]. The advantage to this approach is that the diseased site can be exposed to high concentrations of an agent without high systemic exposure. Below, we will review two of the many agents described for the purpose of combatting infection.

### 2.3. Chlorohexidine

Chlorhexidine is an antimicrobial rinse used to control bacteria in the oral cavity [99]. It is a broad spectrum antimicrobial biguanide compound that works by disrupting the bacterial cell membrane [99,100,101]. It is a positively charged molecule that destroys the integrity of the negatively charged bacterial cell wall causing cell death [102]. In dentistry, it is commonly used in the form of a mouthwash that patients can use at home, but it can also be used in aerosol, gel or spray form [101]. It is often used in the treatment of gingivitis as it is active against both Gram-positive and Gram-negative bacteria [103,104]. However, chlorhexidine does not act as effectively against already formed biofilms and plaques because of the protective EPS layer preventing the chemical from reaching the bacterial cell membrane [105,106]. Though chlorohexidine is effective at reducing gingival plaque and gingivitis, prolonged use for longer than 4 weeks results in extrinsic staining [106,107]. Chlorohexidine is also known to have a temporary effect on taste perception as it blocks the ability to taste salt and is bitter tasting [108,109]. In addition, allergic reactions to chlorhexidine are possible though infrequent [110].

### 2.4. Calcium Hydroxide

Calcium hydroxide is used in endodontics and other areas of dentistry for its ability to induce hard tissue deposition [111,112]. Importantly, it can have an antimicrobial effect, which can help disinfect the root canal system and prevent reinfection of the treated space, which is especially important in the inter-visit timeframe [113]. Calcium hydroxide is also used as a cavity liner when dentists remove deep decay from teeth. Calcium hydroxide creates an alkaline environment that produces its antibacterial effect. The hydroxyl group on calcium hydroxide is responsible for the high pH (12.5), which creates an alkaline environment in the root canal space. The high pH causes a superficial layer of necrotic pulp to form, which protects the tissue beyond from a strong inflammatory response [113]. This high pH environment is also thought to contribute to other biological responses in the pulp cavity space, such as cell differentiation and dentin formation [113,114,115]. Calcium hydroxide paste is injected into the root canal space in paste form with radiopacifiers. While toxic to mammalian cells, it is limited to spaces that have limited contact with living tissue such as the infected/extirpated root canal space.

## 3. Antimicrobial Peptides

**Figure 4:**
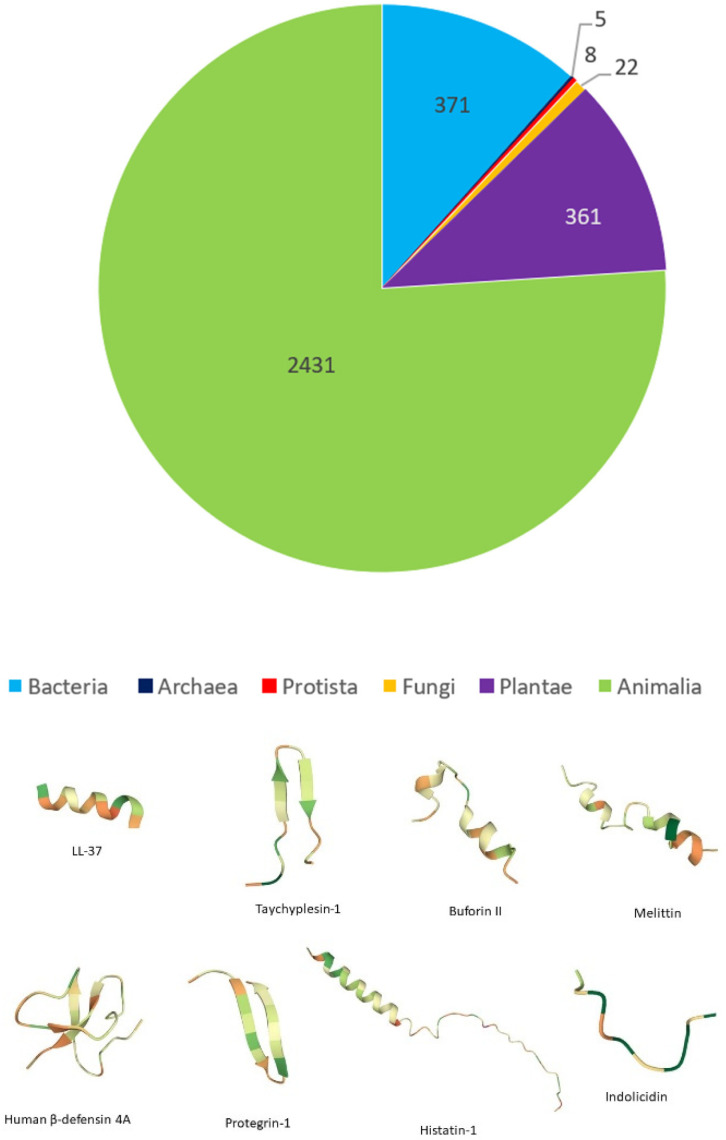
Sources of AMPs from each of the six kingdoms, as of 18 October 2021, and secondary structures of representative AMPs. Data compiled from the Antimicrobial Peptide Database (https://aps.unmc.edu/, accessed on 1 August 2022). Basic residues are shown as orange, dark green represents aromatic residues, light green represents nonpolar residues and proline is shown in yellow. All structures were modeled using UniProt and predicted through either NMR, X-ray, or AlphaFold.

Antimicrobial peptides (AMPs) are a structurally diverse class of small peptides that are active against invasive pathogens [116,117]. Genetically encoded as inactive precursor proteins, AMPs have been isolated from neutrophils and epithelial tissue [118] in a variety of organisms (Figure 4). Many naturally derived AMPs are referred to as host defense peptides (HDPs) for their role in regulating the innate immune response in host cells, particularly in those lacking adaptive immunity [119]. AMPs also display anti-tumor properties in addition to their antibacterial, antifungal and antiviral activity. Due to their nonspecific mechanisms of action with pathogens, AMPs show promise in controlling infections, leading to decreased antibiotic use.

Many AMPs exist as a propeptide before being activated by cleavage of a nonfunctional region, such as buforin II [120]. Glycosylation, phosphorylation, C-terminal amidation and amino acid isomerization are among other post-translational modifications (PTMs) that convert AMPs into fully functional forms [121,122]. With the exception of proteolytically cleaved peptides, low sequence conservation is observed across species, which contributes to their diverse function and activity. Table 1 describes AMPs found in diverse members of the animal kingdom.

Through the inhibition or activation of immune cells [116], some AMPs participate in immunomodulation. By interacting with signaling pathways, they can affect cell proliferation through interference with key aspects of cell division, as well as exhibit pro- and anti-inflammatory properties [123]. This section will include a summary of AMP characteristics and properties. A review of natural and synthetic cationic AMPs will be discussed, as well as patterns of microbial resistance.

### 3.1. Characteristics

#### 3.1.1. Charge

Charge is an important characteristic affecting the activity of cationic AMPs. Through non-specific electrostatic interactions, positively-charged residues associate with the anionic head groups of membrane phospholipids [124]. Compared to eukaryotic cell membranes, bacterial membranes contain charged phospholipid head groups. The coulombic attraction between cationic peptides and anionic phosphate groups results in higher binding specificity for both Gram-positive and Gram-negative bacteria compared to the neutral surface of eukaryotic membranes [125]. The prevalence of cationic amino acids such as arginine and lysine are observed in many AMPs [126]. Additionally, positively charged residues located near the carboxy-terminus may aid AMP insertion into the outer membrane surface. Arg residues in particular have been implicated in increased membrane insertion and translocation, relative to Lys or His, due in part to the ability of guanidinium groups to form hydrogen bonds with the hydrophobic core of the lipid bilayer [127].

#### 3.1.2. Hydrophobicity

The inclusion of specific amino acids is related to a peptide’s hydrophobicity, which determines the ability to partition from the aqueous extracellular matrix into the lipid bilayer of the cell membrane [128]. Partitioning across the membrane interface, a dynamic juncture without a sharp separation between the aqueous extracellular space and hydrocarbon core, is a complex phenomenon that relies on hydrophobic effects, which are characterized in terms of enthalpy and entropy [128,129].

The insertion of AMPs into the inner core of the membrane’s lipid bilayer is influenced heavily by hydrophobic interactions between residues such as proline and tryptophan with hydrocarbon phospholipid tails. The positioning of these residues near the inner section of an AMP can lead to further insertion into the nonpolar core of the membrane [130,131]. This spontaneous phenomenon is a result of entropic effects and further stabilizes peptide secondary structure through hydrogen bonds between charged side groups of residues with surrounding water molecules [132].

The hydrophobicity of a peptide is directly correlated to its activity. A more positive value on hydrophobicity scales, such as the statistical-based Zviling [133] or Wimley–White [134] scales, is observed with higher membrane permeabilization. However, it comes at a cost—namely, increased cytotoxicity to host cells due to nonspecific interactions of insoluble, highly hydrophobic AMPs with membranes, regardless of composition [135,136,137].

Hydrophobicity is also impacted by the initial electrostatic binding of AMPs. Membrane studies on indolicidin derivatives [138] suggest that permeabilization of the bilayer is not simply a combination of electrostatic and hydrophobic interactions. Instead, the influence of electrostatics has an inverse correlation on membrane insertion, with observed effects on AMP solvation.

#### 3.1.3. Amphiphilicity

While the hydrophobicity of AMPs is an important factor in its ability to permeabilize cell membranes, the placement of these hydrophobic regions is integral to the connection between AMP structure and activity. Amphiphilicity, the presence of separate polar and nonpolar regions [139], is key to antimicrobial activity. The hydrophobic moment (µH) [140] of a peptide is the vector sum of hydrophobicity and provides a way to measure amphiphilicity in α-helices. Its significance can be thought of as a combination of the impact of net charge and hydrophobicity—a careful balance between each of these measurements is necessary for the efficacy of an AMP. To study the effects of amphiphilicity and hydrophobicity on membrane partitioning, Fernández-Vidal et al. measured the Gibbs free energy of AMPs, transitioning from their unfolded state in aqueous solutions to an α-helix at the membrane interface [141]. Using non-charged residues in the design of 6 synthetic AMPs, peptide amphiphilicity was varied while hydrophobicity was kept constant. µH was observed to be inversely proportional to the free energy of partitioning, suggesting that an increase in this parameter would correlate to higher membrane interactions. Interestingly, asymmetrical amphiphilicity is observed in AMPs with high antimicrobial activity and low host toxicity [142], reinforcing the inverse relationship between cell selectivity and hydrophobicity.

#### 3.1.4. Structure

The secondary structure of AMPs depends upon interactions in the peptide backbone, as well as partitioning–folding coupling [143]. AMPs are disordered in aqueous solutions and most adopt an α-helical or folded conformation (containing antiparallel β-sheets) in membrane-mimetic environments. Relative to β-sheet-containing AMPs, there are little to no cysteine residues found in α-helical peptides, such as magainins [144]. The disulfide bridges present in β-sheet-containing AMPs are a fundamental architectural element in peptide structure and are the only naturally occurring intra-peptide covalent bond. Formed by the oxidation of thiol groups between two cysteine residues, disulfide bridges stabilize peptide secondary and tertiary structure. Also referred to as cystine residues, disulfides are often formed in vivo via thiol-disulfide exchange reactions, where a disulfide bond is transferred from a linked pair of Cys to a reduced thiol [145]. Despite their increased stability relative to other side-chain interactions, disulfide bridges are susceptible to cleavage in the presence of reductants. It is interesting to note the prevalence of aromatic amino acids near Cys residues [146].

### 3.2. Mechanisms of Action

The mechanisms of action of AMPs are concentration dependent. At low concentrations, they are oriented parallel to the surface of the lipid bilayer. However, perpendicular assembly within the hydrocarbon core of the bilayer is observed with high AMP concentration, forming transmembrane pores [147].

**Figure 5:**
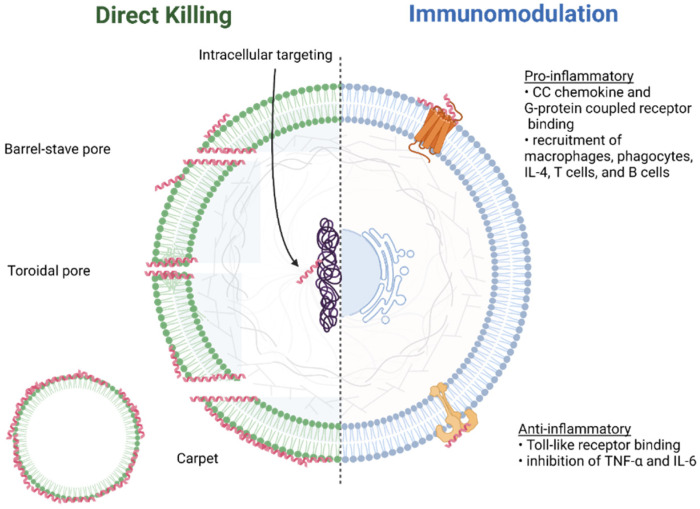
Overview of roles and observed mechanisms of action of antimicrobial peptides (created with BioRender.com, accessed on 1 August 2022). Either direct killing of bacteria and fungi, immunomodulation of host cells or a combination of both mechanisms are observed with AMPs.

There are numerous models (Figure 5) used to describe the formation of these transmembrane pores [116,117]. The barrel-stave pore model [148] is the result of interactions of the hydrophobic regions of the peptide with the hydrocarbon core of the lipid bilayer. Toroidal pores [149], on the other hand, are formed through the attraction between the hydrophilic region regions of the peptide with the charged phospholipid heads on the surface of the membrane. The carpet model [150] is associated with the disruption of the cell membrane through the formation of micelles and is observed to be concentration-dependent. Membrane depolarization describes the process of electroporation [151], where pores are formed due to changes in the external electric field of the membrane.

These models may not fully account for microbial killing. Other phenomena observed may provide a more accurate picture of the mechanisms of action, particularly with fungal infections. For instance, buforin II has been observed to interact with cells via membrane translocation [152]; in other words, cell penetrating. Tachyplesin also demonstrates cell-penetrating properties and can deliver macromolecular cargo to nuclei via non-endocytic modes [153].

Additionally, AMPs can stimulate or reduce host inflammatory response. Sublancin, an AMP derived from *Bacillus subtilis*, promotes the methicillin-resistant Staphylococcus aureus (MRSA)—induced production of interleukin-6 (IL-6) and monocyte chemoattract protein-1 (MCP-1) at 24 h post—infection in murine peritoneal cells, while a lower concentration of tumor necrosis factor-alpha (TNF-α) is seen with the addition of the AMP [154]. Neutrophils (polymorphonuclear leukocytes) are commonly observed to act in the innate immune response to pathogens. This process involves adherence to pathogens followed by phagocytosis or through the release of AMP-containing granules. Despite the ineffectiveness of physiological concentrations of AMPs in antimicrobial killing in healthy human gingiva, they are found in overabundance in the epithelia of patients with periodontitis [155]. Interestingly, a locus near DEFA1A3 has been implicated in aggressive and, to a lesser degree, severe chronic periodontitis in German, Dutch and Turkish samples in a genome-wide association study (GWAS). Because the mechanisms involved in the immune response to oral diseases are as diverse as the various disease phenotypes, the identification of genetic risk factors is a significant step in further exploration of the role of AMPs in signaling pathways [156]. Additionally, single nucleotide polymorphisms (SNPs) and the overexpression of toll-like receptor (TLR) genes correlate to decreased hBD-2 production, although the direct link between these processes remains unclear [157].

**Figure 6:**
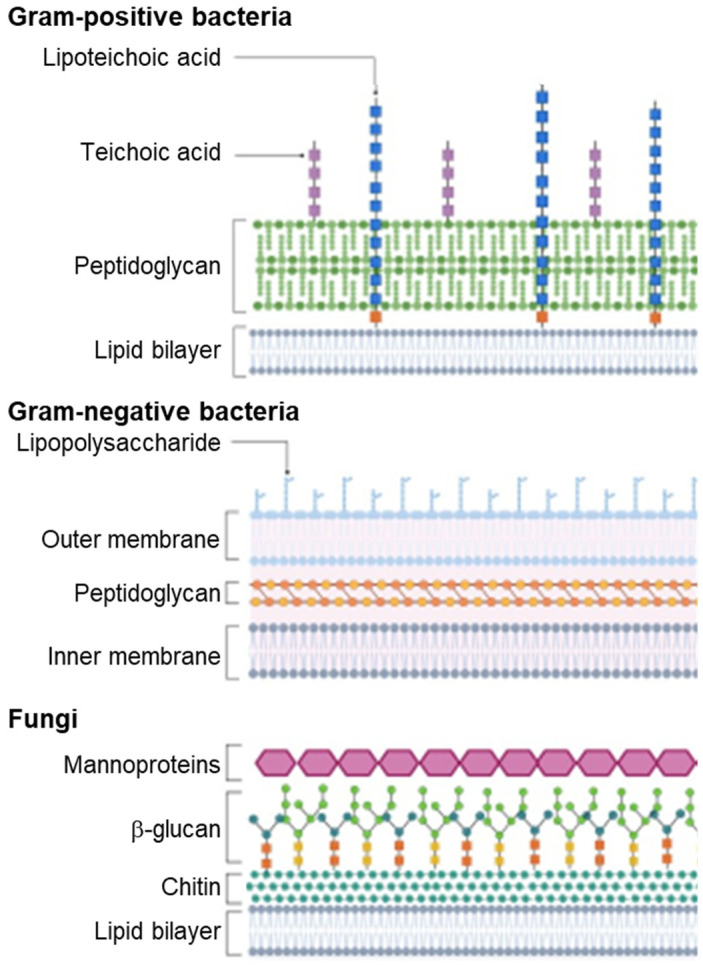
Comparison of fungal and bacterial cells (created with BioRender.com, accessed on 1 August 2022). Gram-positive bacteria and fungi contain glycan layers on the extracellular side of the lipid membrane, while a second membrane encapsulates the peptidoglycan layer in Gram-negative bacteria.

The lipid composition of the outer membranes of Gram-negative bacteria accounts for the preferential targeting of AMPs [136]. Initial interaction depends upon the strength of the binding between AMPs and outer surfaces (Figure 6). Negatively charged phospholipid heads and lipopolysaccharides (LPS) found on the outer membrane participate in electrostatic interactions with the hydrophobic regions of the peptides. These interactions are also observed with the lipoteichoic acids (LTA) and peptidoglycans that comprise the cell wall of Gram-positive bacteria. Weaker hydrophobic interactions are also observed between AMPs and bacterial surfaces, albeit to a lesser extent than with eukaryotic membranes [158].

Furthermore, differences between the lipid structures that comprise bacterial and eukaryotic membranes may explain the specificity of AMPs. While both cells contain anionic phosphate head groups, the prevalence of lipids with a specific geometry influences peptide binding. For instance, bacterial membranes contain lipids such as phosphatidylserine (PS) that have less bulky substituents than those primarily found in eukaryotes, such as phosphatidylcholine (PC), which can interfere with AMP binding.

### 3.3. Classification Based on Source

Due to their diversity, AMPs can be categorized into various subclasses, based on origin, structure, amino acid composition, mechanism of action and activity. Structure and mechanisms of action have already been discussed in Section 2 and Section 3, respectively; the next subsections will focus on the remaining categories of classification.

#### 3.3.1. Origin

Antimicrobial peptides have been isolated from a multitude of species, including prokaryotes and eukaryotes. The following sections will focus on animal-derived sources of AMPs; comprehensive assessments of AMPs derived from other biological organisms have been previously reviewed [124,125,158]. The three major classes of mammalian-derived AMPs are cathelicidins, defensins and histatins, each of which are significant in establishing and protecting oral health.

#### 3.3.2. Cathelicidins

First isolated from bovine neutrophil lysates [159], cathelicidins comprise a class of peptides that contain a conserved N-terminal region and a variable C-terminal region. They are primarily stored in granulocytes and play an important role in the regulation of innate immunity. Over 70% of cathelicidins’ N-terminal sequence is homologous with cathelin, a cathepsin L inhibitor. Cathelicidin-encoding genes produce a precursor, e.g., human CAP-18 [118]. Cleavage of this propeptide yields cathelin and any of a number of cathelicidin peptides [160,161]. The lack of homology among residues in the C-terminus account for the structural diversity of cathelicidins, which includes helical, linear and folded arrangements, depending on the residue composition. To date, only one cathelicidin has been isolated in humans—LL-37. Molecular dynamic and crystallography studies demonstrate that LL-37 forms a tetramer comprised of antiparallel dimers in the presence of detergents and bacterial membranes [162] to form peptide-lined channels that are stabilized by hydrogen-bond interactions between its interfaces and PE/PG structures. Despite its success in inhibiting clinically relevant bacteria at low concentrations, LL-37 is unable to disrupt any of the stages of biofilm formation up to concentrations of 25 µM [163].

#### 3.3.3. Defensins

Defensins are cysteine-rich peptides characterized by the presence of two to three disulfide bonds that form β-sheets. They are primarily classified based on their disulfide bond orientation. These superfamilies, *cis*- and *trans*-defensins, are evolutionarily divergent from each other and participate in both direct microbial killing and immunomodulatory pathways. The presence of disulfide bonds imparts defensins with increased resistance to proteolysis. *Trans*-defensins are mostly found in vertebrates and contain β-sheets that connect to two structural motifs due to antiparallel disulfide bridge pairs [164]. AMPs that comprise this superfamily are classified as either α-, β- or θ-defensins. Unlike the structure of the others, θ-defensins are cyclic peptides with a β-hairpin motif. *Cis*-defensins, on the other hand, are found in plants, fungi and invertebrate animals across many phyla. They contain parallel disulfide bonds that link β-strands to one other structural motif.

#### 3.3.4. Histatins

Histatins, named for prevalence of histidine residues, play a significant role in wound healing. Primarily found in human and simian saliva, these AMPs are either encoded by HIS1 and HIS2 (histatin 1 and histatin 3, respectively [165]), proteolytically cleaved or otherwise modified following translation. Histatin 5, processed from histatin 3, displays the strongest antimicrobial activity [166] and is effective in treating oral candidiasis [167] and inhibiting *C. albicans* biofilm adherence in mucosal epithelia [168]. Conformational studies reveal that Hst-5 is stabilized through the binding of histidine residues to zinc and copper [169], which is also correlated to increased antimicrobial activity.

### 3.4. De Novo Peptides

Strategies to improve upon the antimicrobial activity of these peptides have included the design of de novo AMPs. The purpose of the rational design process focuses on further elucidation of mechanisms of action, such as the extent of membrane disruption, or probing the relationship between structural elements of the peptide and its activity. This is even more necessary given the relative cost of solid phase peptide synthesis as compared to other antimicrobial drugs [170] and occasional need for high concentrations in natural forms [155]. Common synthetic strategies include:

1. Cyclization of linear regions;
2. D-amino acid substitution to take advantage of enzyme-substrate specificity to evade protease recognition and subsequent degradation;
3. Replacing hydrophobic residues studies the effects of hydrophobicity and amphiphilicity on cytotoxicity.

Melittin, a well-studied AMP derived from bee venom, has served as a starting point for many of these studies. Compared to other AMPs, melittin displays antimicrobial activity across a broad spectrum of pathogenic bacteria and fungi. However, high hemolysis has also been observed, prompting investigation into how this peptide can be modified to inhibit microbes with low cytotoxicity to host cells. In one study, researchers analyzed the overall hydrophobicity of melittin through comparison to analogues [171]. Lower hemolytic activity and comparable MIC/MBC (minimum inhibitory concentration/minimum bactericidal concentration) values against *S. aureus*, *E. coli* and *P. aeruginosa* were observed with substitution and/or deletion of hydrophobic residues for tryptophan.

Derivatives of indolicidin, found in bovine neutrophils, have also been studied in vitro. Comparison of two novel peptides demonstrate that a decrease in hydrophobicity and increase in hydrophobic moment and net charge correlate to higher antibacterial activity and lower cytotoxicity. However, differences in the membrane permeabilization between Gram-positive and Gram-negative bacteria may result from electrostatic interactions from cell wall [172].

Modifications to the 3D assembly of AMPs have been explored recently. Structurally nanoengineered antimicrobial peptide polymers (SNAPPs) are antimicrobial micelles or vesicles that share many characteristics with natural AMPs, most notably the inclusion of cationic residues [173]. However, the peptide “arms” of SNAPPs allows for enhanced interaction between with bacterial membranes. The formation of these structures typically relies on self-assembly through ring-opening polymerization (ROP) strategies. Although they demonstrate antimicrobial activity in bacterial models, their efficacy in biomimetic environments is altered. The presence of divalent cations disrupted the activity of SNAPPs prepared by Lam et al., although the addition of a chelating agent mitigated this effect [174]. On the other hand, the presence of serum proteins reduces cytotoxicity, which is correlated to decreased interaction with host immune cells [175]. Additionally, dimerization, or the process of linking two monomers to form a dimer, is associated with increased antimicrobial activity against MDR clinical pathogens [176].

To create novel alternatives to antibiotics, some design strategies have been influenced by another class of microbe: viruses. Recently, the structure of severe acute respiratory syndrome coronavirus-2 (SARS-CoV-2) has been adapted to create a lipopeptide delivery system, called SNALs (surface-nanoengineered antimicrobial liposomes) [177]. Mimicking the SARS-CoV-2 spike protein, SNALs are able to fuse to bacterial surface receptors. Observed mechanisms include perturbation of the cell membrane and intracellular targeting. Unique peptide scaffolds, such as β-hairpin hydrogels, have also been investigated for wound healing properties [178]. Additionally, biomimetic polymers synthesized by Tsukamoto et al. have demonstrated disruption of unilamellar vesicles [179]. These methacrylate-backed polymers, with a net positive charge, contain a combination of ammonium and hydrophobic groups as side chains and display MIC and hemolytic values to natural AMPs.

### 3.5. AMP Resistance

The rise in multidrug resistance (MDR) among opportunistic pathogens is intrinsically related to overexposure to traditional antibiotics, both in nosocomial and agricultural environments. Simply put, resistance is the ability of a microbe to tolerate treatments designed to inhibit or kill. Despite the moderate success of AMPs in inhibiting pathogenesis, resistance to these peptides have also been observed. Common resistance mechanisms involve point mutations in bacterial genes encoding for proteins essential in lipid synthesis and maintenance of membrane integrity. Resistance is typically observed following repeated treatment, but application of selective pressure (pH, temperature, salt concentration) can lead to decreased susceptibility.

MprF, or multiple peptide resistance factor, is an integral membrane-bound enzyme with bifunctionality—it catalyzes the transfer of lysine and alanine residues to phosphatidylglycerol (PG) and cardiolipin (CL) as well as translocates the lysl- and alalyl-modified lipid structures from the cytosol-facing side of the lipid bilayer to the outer surface. Point mutations in the binding site of the synthetic domain of MprF is related to the upregulation of Lys- or Ala-PG [180]. This is associated with a decrease in AMP binding, due to the introduction of positive charge by Lys and Ala to the outer membrane. Additionally, resistance to polymyxin B, an AMP that binds to lipopolysaccharides (LPS) on Gram-negative bacteria, has been observed in Enterobacteriaceae (e.g., *Salmonella enterica* [181,182]) and *Pseudomonas aeruginosa* [183].

In addition to target mutations and upregulation of efflux pumps, an increase in protease activity is also observed in resistance mechanisms. For example, studies conducted on gingival and subgingival tissue indicate an inverse correlation between human β-defensin 2 (hBD-2) and *P. gingivalis*-associated protease activity in periodontitis patients, emphasizing the importance of peptide design strategies that are resistant to microbial proteases [184]. Furthermore, epithelia from different physiological regions induce hBD-2 through various signaling pathways. LPS in bacterial extracellular matrices stimulate mRNA transcription of hBD-2 in the trachea, while it was not observed to significantly affect hBD-2 production in oral epithelia [185]. This could be explained by the prevalence of commensal bacteria in the oral cavity, where the use of LPS as a stimulant would result in an overabundance of hBD-2 and loss of viable cells. A more thorough understanding of the various distinct pathways used to regulate hBD-2 production can inform a more targeted approach to the design of synthetic AMPs for treating oral diseases.

Recently, modifications in metabolism-associated genes have been detected in clinical pathogens that have been treated with traditional antibiotics [186]. Changes in the metabolic activity of AMP-treated microbes may provide further insight into the resistance pathways that pathogens use to avoid inhibition. Cell death is associated with accumulation of reactive oxygen species (ROS) after treatment with traditional antibiotics [187], so AMPs that result in ROS accumulation that may lead to decreased resistance.

## 4. Conclusions and Future Directions

A major impediment in screening for undiscovered natural or synthetic AMPs is fully understanding how microbial pathogens interact with their hosts’ cells. To this end, machine learning has been employed to allow researchers to investigate the contributing factors behind virulence, pathogenicity and host-immune response. SweetTalk, a language-based model that utilizes a bidirectional recurrent neural network (RNN), has recently been developed to study glycan-mediated interactions between bacteria and host cells [188]. Through the modeling of glycan assembly patterns, bacterial virulence and its effect on host immune response can be predicted.

Another obstacle in making more AMPs clinically approved is their low bioavailability. Compared to small molecule drugs, AMPs are relatively larger and require higher concentrations in order to have an appreciable effect. Wang et al. recently used gene sequencing to discover a novel small molecule antimicrobial, macolacin, identified through biosynthetic gene clusters in colistin-resistant bacteria [189].

Simulation-based approaches such as atomic detail molecular dynamic modeling [190] can provide information about key structural motifs for peptide folding and activity that are found in naturally occurring AMPs. De novo syntheses can also utilize molecular grafting techniques, where small biomolecules act as substrates for AMP functionalization. Plant-based cyclotides have been used as a scaffold for a short synthetic AMP, with low MIC values observed for clinically relevant Gram-positive and Gram-negative bacteria [191]. Furthermore, a combination of peptide sequencing and electron-transfer dissociation (ETD) mass spectrometry can be used to analyze post-translational modifications (PTMs) in natural AMPs that are applicable to novel peptide design [192].

AMPs and other novel antimicrobials can fulfil two important functions in controlling oral diseases:

1. Modify the composition of biofilms on tooth, soft tissue and prosthetic surfaces, the goal being to reverse dysbiosis without destroying the commensal flora. Novel AMP-inspired antimicrobials have also been investigated as ways to reduce *Candida* growth on denture surfaces [193]. It is conceivable that a healthy individual can use biofilm-modifying AMPs as part of a preventive regimen.
2. Treat endodontic infections and deep caries by eliminating bacterial populations from the normally sterile interior of the tooth (dentin and pulp space). In this application, AMPs would be applied as part of the professional treatment of dental infections [194,195].

## Figures and Tables

**Table 1:** Representative examples of naturally occurring AMPs. Secondary structure refers to the observed structure in membrane-mimetic environments. * Net charge calculated at physiological pH (pH = 7.4). While some of the AMPs contain anionic residues, they display a net positive charge.

Example	Primary Structure	Secondary Structure	Origin	* Net Charge
Melittin	GIGAVLKVLTTGLPALISWIKRKRQQ-NH_2_	α-helix	*Apis mellifera*	+6
Buforin II	TRSSRAGLQFPVGRVHRLLRK	α-helix	*Bufo gargarizans*	+6
LL-37	LLGDFFRKSKEKIGKEFKRIVQRIKDFLRNLVPRTES	α-helix	*Homo sapiens*	+6
Protegrin-1	RGGRLCYCRRRFCVCVGR	β-sheet	*Sus domesticus*	+6
Tachyplesin-1	KWCFRVCYRGICYRRCR	β-sheet	*Tachypleus tridentatus*	+6
Human neutrophil peptide-1	ACYCRIPACIAGERRYGTCIYQGRLWAFCC	β-sheet	*Homo sapiens*	+3
Indolicidin	ILPWKWPWWPWRR-NH_2_	Random coil	*Bos taurus*	+4
Histatin-5	DSHAKRHHGYKRKFHEKHHSHRGY	Random coil	*Homo sapiens*	+6

## Data Availability

Not applicable.
